# Exploring the structural changes on excitation of a luminescent organic bromine-substituted complex by in-house time-resolved pump-probe diffraction

**DOI:** 10.1063/1.4978240

**Published:** 2017-03-09

**Authors:** Krishnayan Basuroy, Yang Chen, Sounak Sarkar, Jason Benedict, Philip Coppens

**Affiliations:** Department of Chemistry, University at Buffalo, State University of New York, Buffalo, New York 14260-3000, USA

## Abstract

The structural changes accompanying the excitation of the luminescent dibromobenzene derivative, 1,4-dibromo-2,5-bis(octyloxy)benzene, have been measured by in-house monochromatic time-resolved (TR) diffraction at 90 K. Results show an increment of the very short intermolecular Br•••Br contact distance from 3.290 Å to 3.380 Å. Calculations show the Br…Br interaction to be strongly repulsive in both the Ground and Excited states but significantly relaxed by the lengthening of the contact distance on excitation. The stability of the crystals is attributed to the many weak C-H···Br and C-H···π intermolecular interactions. The study described is the first practical application of In-House Time-Resolved diffraction, made possible by the continuing increase in the brightness of X-ray sources and the sensitivity of our detectors.

## I. INTRODUCTION

**FIG. 1.**
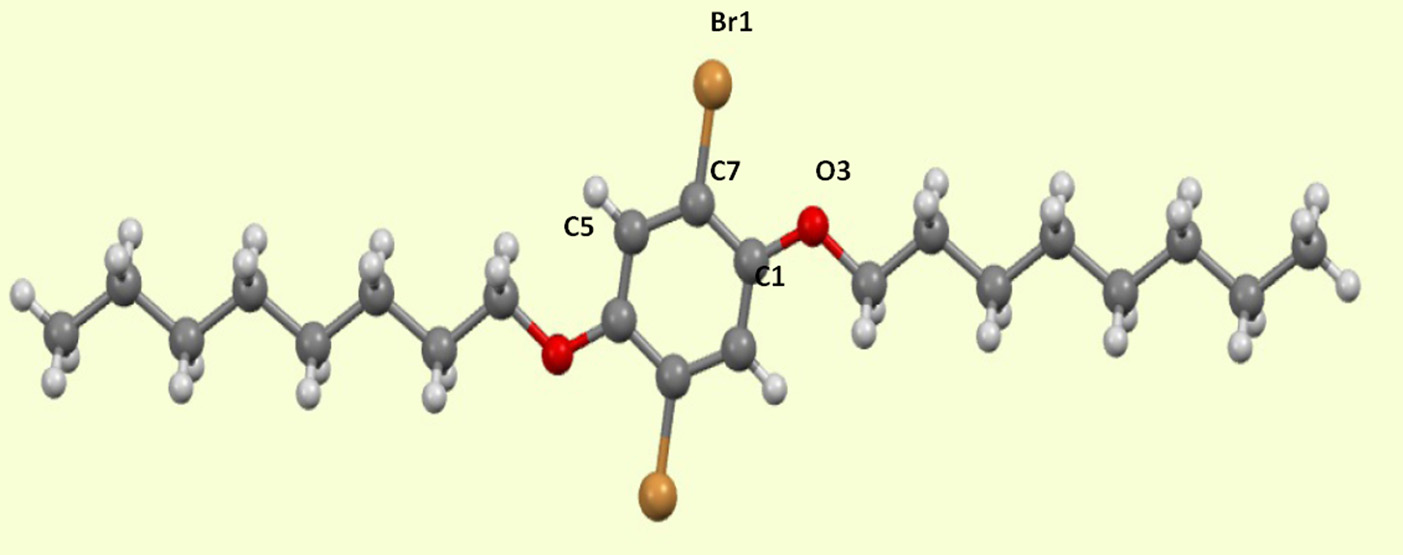
Molecular structure of PhBr_2_(O(CH_2_)_7_(CH_3_))_2_ and labeling of atoms.

It has long been accepted that highly luminescent materials require the presence of heavy atoms such as Pt, Ir, Re, or rare earth metals, whereas organic molecular crystals show short ambient temperature phosphorescent lifetimes because of non-radiative deactivation and triplet-triplet deactivation. But more recently organic luminescent solids have received widespread attention; it has been shown that careful molecular design can be used to synthesize organic solids with phosphorescence lifetimes of milliseconds and longer (Mukherjee and Thilagar, 2015; An *et al.*, 2015, Gong *et al.*, 2015; and Shi *et al.*, 2016). The solids are typically based on Br containing molecules with short Br···Br interactions. Shi *et al.* conclude that the enhancement of phosphorescence results from increased heavy atom interactions in the organic crystals. Four complexes were included in that study labeled PhBr_2_(O(CH_2_)_5_(CH_3_))_2_ (**I**), PhBr_2_(O(CH_2_)_7_(CH_3_))_2_ (**II**, Fig. 1) with only one Br···Br interaction per molecule, and PhBr_2_(O(CH_2_)_6_)Br)_2_ (**III**) and PhBr_2_(O(CH_2_)_8_)Br)_2_ (**IV**) each with three Br···Br interactions per molecule due to the participation of Br atoms at the end of the alkyl chains in the Br···Br interactions. Room-temperature lifetimes and quantum yields for the four compounds were reported to be τ = 8.3, 6.7, 6.5, and 6.4 ms and φ_phos_ = 3.4%, 8.9%, 21.9%, and 13.1%, respectively.

Notwithstanding the considerable attention concentrated on organic luminescent solids, the structural changes occurring on excitation are not known. Because of their long lifetimes, the compounds are eminently suited for study by the Pump-Probe In-House Time Resolved Diffraction (IHTRD), a technique we have developed recently (Kaminski *et al.*, 2014 and Trzop *et al.*, 2014). Compound **II** shows the shortest room temperature Br**^…^**Br distance of 3.380 Å in the group (Shi *et al.*, 2016), which is much below the nominal Br…Br contact distance of 3.70 Å. This compound is the subject of the current study.

## II. EXPERIMENTAL

### A. Synthesis and spectroscopic measurements

**FIG. 2.**
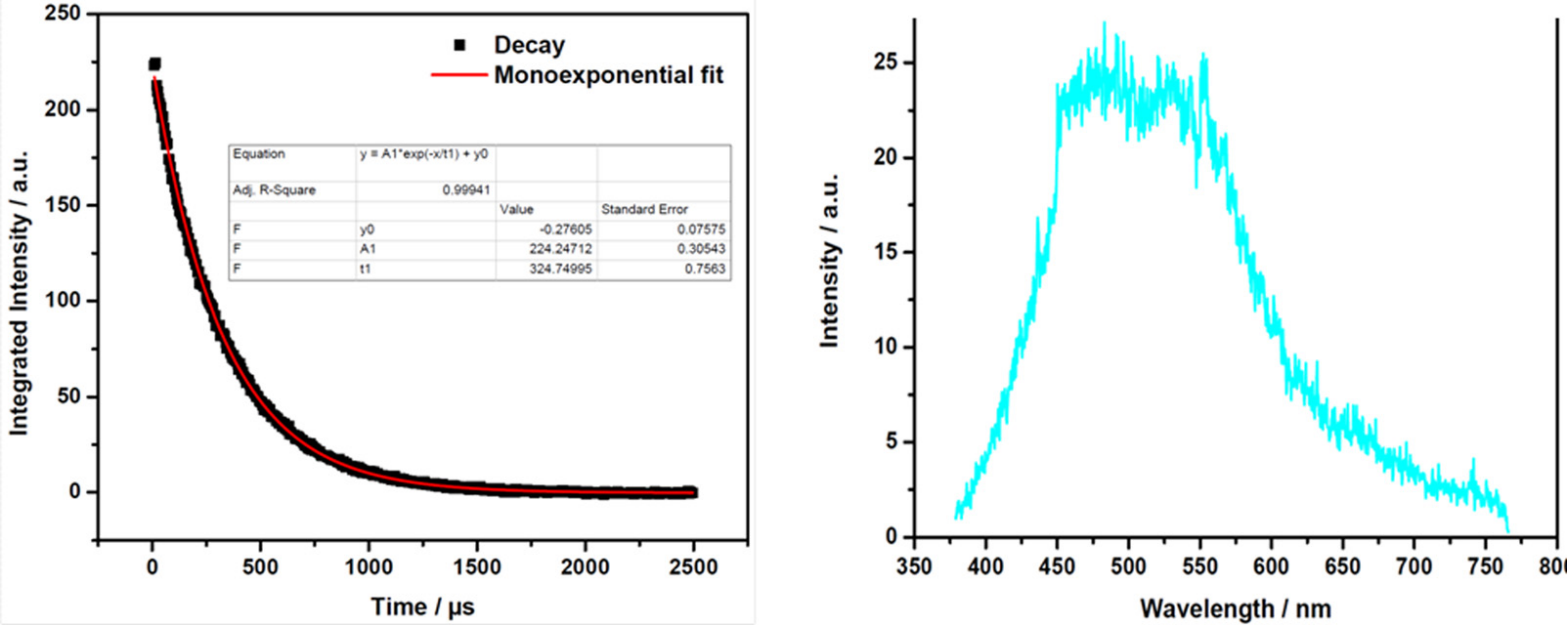
(a) 90 K lifetime of crystals of II. Excitation 355 nm. (b) Luminescence of II. Excitation 355 nm.

Synthesis of PhBr_2_(O(CH_2_)_7_(CH_3_))_2_ was carried out according to Shi *et al*. (2016). The lifetime and emission spectra of the excited state (ES) species were measured on single crystals at 90 K. The phosphorescent lifetime at 325 *μ*s of (**II**) (Fig. 1(a)) may be compared with the reported lifetime of 6.7 ms at ambient temperature, which however was not reproduced in our experiments. The observed emission peak at 492 nm (Fig. 1(b)) is in reasonable agreement with the literature (Shi *et al.*, 2016). Further details are given in Section SI-1 of the supplementary material (Fig. 2). The 90 K lifetime measurement results and the luminescent spectrum of II are shown in Figs. 2(a) and 2(b), respectively.

### B. Static and time-resolved X-ray crystallography

**FIG. 3.**
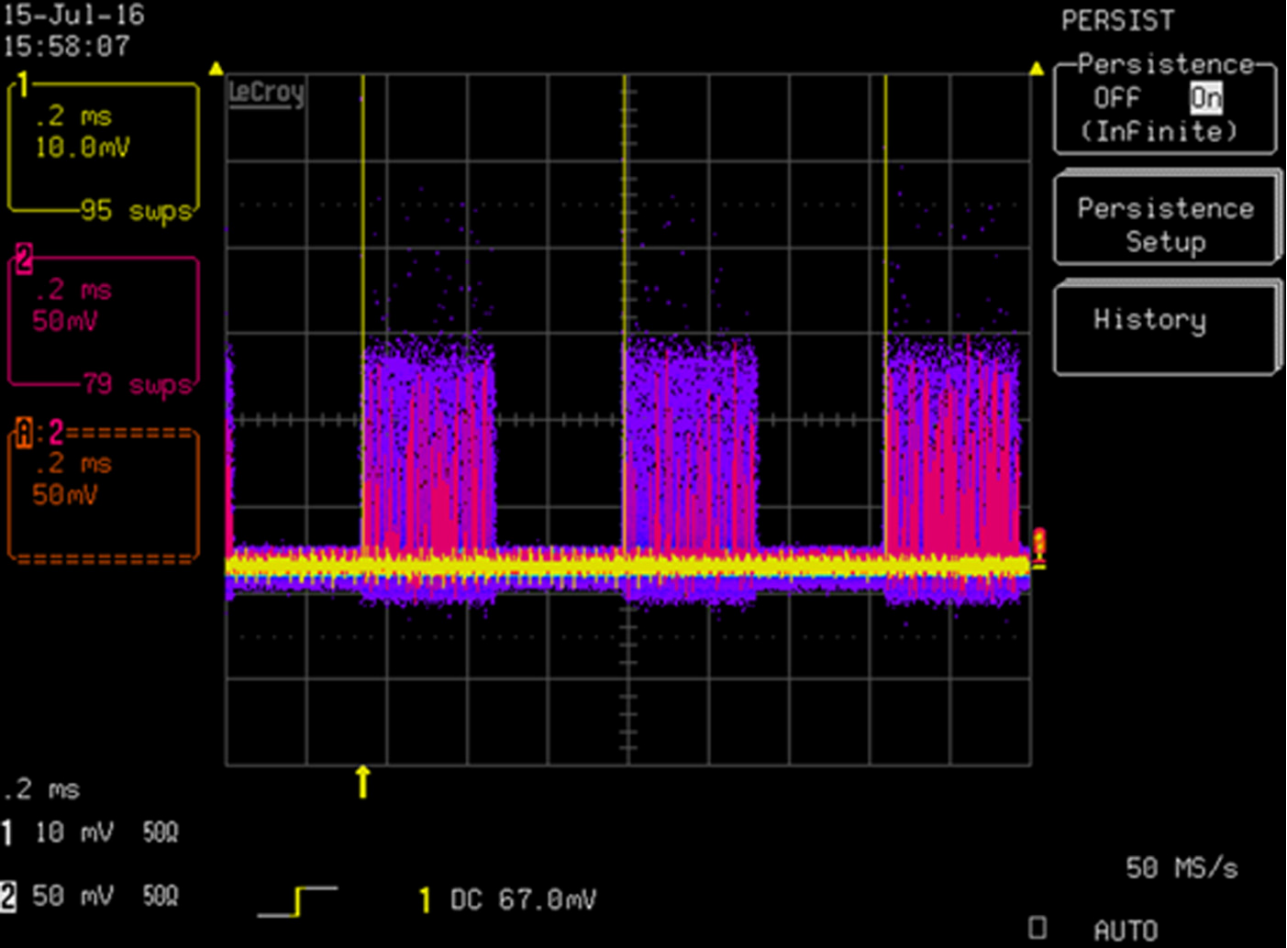
Oscilloscope view showing the X-ray period (purple) and the synchronized laser pulses (yellow) during the pump probe stage of the experiment.

Ground state (GS) data sets were collected at 90 K on a Turbo-X Bruker Rotating Anode diffractometer equipped with Helios multi-layer optics (see Section SI-2 of the supplementary material for details). In order to provide the pulsed X-ray beam required for the Pump-Probe experiments, an optical chopper was installed in the X-ray beam (Kamiński *et al*., 2014). A 30 slot chopper was used at 103 rps to give a pulse length of about 325 *μ*s matched to the phosphorescent lifetime of the current luminophore. The laser pulse repetition rate was adjusted to 1.538 kHz corresponding to a repeat of 650 *μ*s to match the repeat rate of the X-ray pulses. 355 nm radiation from a tripled Nd-YAG laser with an 8.82 *μ*J energy per pulse was used. The laser pulses were synchronized with the X-ray chopper to produce a laser pulse prior to every X-ray exposure, as illustrated in the cathode ray image of Fig. 3. For each φ-setting, data were collected 10 times for both laser-ON and laser-OFF measurements to allow proper evaluation of the statistical uncertainties and calculation of the ratios and their standard deviations. A φ range of φ = 0–180° at a fixed value of ω was covered with a Δφ step of 2°. Full details are described elsewhere (Kalinowski *et al.*, 2012). The program MONOUTIL, which is a modified version of LAUEUTIL (Kalinowski *et al*., 2011; 2012), was used in the analysis.

**FIG. 4.**
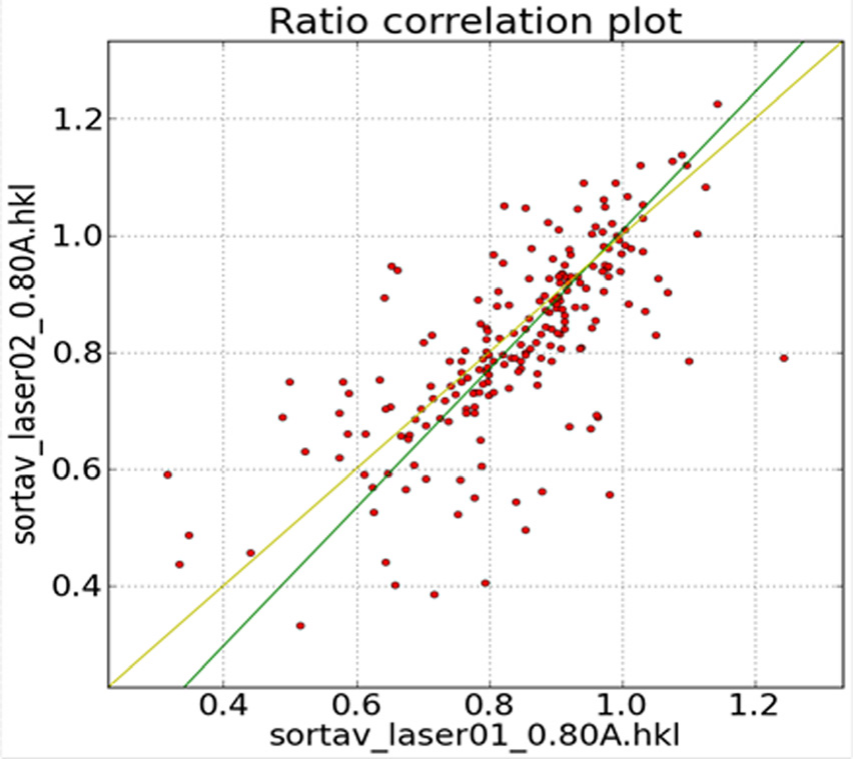
Correlation of TR response ratios between data sets 1 and 2. The same laser powers were used for both data sets. (Reproduced with permission from Coppens *et al*. 2017, Copyright 2017 International Union of Crystallography.)

The first two sets of the six sets collected are very short. They were measured prior to the start of more extensive data collection in order to establish the existence of a response to the laser-light exposure and the reproducibility of the measurements (Coppens *et al.*, 2017). The correlation between the ratios measured in these sets is reasonable, as shown in Fig. 4.

Subsequent sets, laser03–06, collected with different sample crystals, were scaled and merged separately by the SORTAV program (Blessing, 1987; 1995; 1997). Subsequently, a combined scaled/merged data set was obtained by scaling of the individual data sets by a scale factor *k*(*η*)_set_ = ⟨|*η*|⟩_all_ / ⟨|*η*|⟩_set_; in which ⟨|*η*|⟩_all_ = the average over all the measured reflections and ⟨|*η*|⟩_set_ = the averages over all measured reflections in a specific data set (Makal *et al.*, 2011). The combined scaled data sets were then merged using the SORTAV program. The combined dataset has 84.0% overall completeness with 1951 unique reflections. The individual data sets are summarized in Table I.

**TABLE I.** Details of data sets.

Crystal size (*μ*m)	Datasets	Laser ON/OFF cycles	X-ray exposure time (s)	Number of frames collected	Goniometer angle ω (°)
≈100 × 90 × 39	Laser01	10	10	600	−145
≈100 × 90 × 39	Laser02	10	10	600	−145
≈72 × 68 × 31	Laser03	10	13	1800	−160
≈68 × 60 × 30	Laser04	10	13	1800	−145
≈65 × 60 × 30	Laser05	10	13	1800	−130
≈76 × 64 × 60	Laser06	10	14	1800	−115

## III. EXPERIMENTAL RESULTS

### A. Ground state structure

As described by Shi *et al.*, the ground state structure of PhBr_2_(O(CH_2_)_7_(CH_3_))_2_ (**II**) consists of sheets of linear molecules connected at ambient temperature by Br···Br bonds at the distance of 3.380 Å, significantly shorter than twice the Br van der Waals radius of 1.90 Å. Our analysis shows this distance to be 3.290 Å at 90 K. The C-Br···Br-C-interaction has the geometry of a type I interaction across a center of symmetry (Desiraju and Parthasarathy, 1989). The four atoms are coplanar with a C-Br···Br angle of 154.7°. Such type I interactions are classified somewhat repulsive based on the charge density of a number of Cl substituted hydroxypyridine and quinolone complexes (Venkatesha and Guru Row, 2010). However, it must be noted that in the complexes described in that study, the type I trans complex shows a larger chlorine-chlorine distance 3.5747(2) Å than those corresponding to the type II interaction with the L geometry (unequal angles of 0° and 90°), both being longer than the Br···Br distance in the present compound. However, in those compounds other strong intermolecular interactions are also present.

Calculated GS interaction energies are repulsive for all basis sets considered. The values are strongly dependent on the type of calculation but are smallest at 0.96 kJ/Mole with the 6-311++G(3df, 3pd) basis set and the PBE0 functional. The crystal structure consists of sheets of the basically linear molecules stacked at distances of 3.5–4.5 Å between the heavier atoms (and less when the hydrogen atoms are taken into account). The sheets, formed by unit-distance translated molecules along the crystallographic “*b*” axis, are widely spaced with interatomic distances of 7–8 Å, except for the very short Br···Br contacts. To explain the stability of the crystals of PhBr_2_(O(CH_2_)_5_(CH_3_))_2_ (**II**) it would appear that the Br···Br interactions are attractive. However, this is not confirmed by our quantum-mechanical calculations discussed below. It is of interest that the literature contains a number of Br···Br contacts in the 3.3–3.4 Å range (see for example, Han *et al*., 2011 and Kitamura *et al*., 2007).

In 1985, Williams and Hsu (Williams and Hsu, 1985) proposed additional interaction terms to explain the stability of layer structures of forms of diatomic halides, which show L-type arrangement linking the diatomic species. In 2010, Nelyubina *et al*. (2010) noted strong I···I interactions in a co-crystal of N-methylpyrazine iodide with I_2_, attributed to a strong influence of molecular interactions on the density of the iodine atoms. None of these explanations seem applicable to the current case in which the Br atoms are well isolated and linked with a type I geometry. On the other hand, the GS structure includes a number of weak **C**-H…**Br** (3.8–4.0 Å), **H**…**Br** (∼3.0 Å), and C-**H**…**π** (∼2.8 Å) inter-sheet intermolecular interactions (distances between boldly marked atoms, H-positions extended to neutron determined values, π interactions measured to center of aromatic ring.). The stability of the crystals is likely due to the combined effect of such weak interactions. An appropriate analysis summarizing all interactions could be based on Gavezzotti's pixel method (Gavezzotti, 2011), but such an analysis is beyond the scope of this paper. Final agreement factors are listed in Section SI-2 of the supplementary material.

### B. Excited state structure

#### 1. Definition of difference Fourier maps in the ES analysis

*Photodifference maps:* Photodifference maps (Kim *et al*., 2002; Zheng *et al*., 2007; Makal *et al*., 2011; Fournier and Coppens, 2014; and Jarzembska *et al*., 2014) illustrate the light-induced changes in terms of the differences between laser-ON and laser-OFF electron density distributions. They are important for the selection of parameters in the following least squares refinements.

**FIG. 5.**
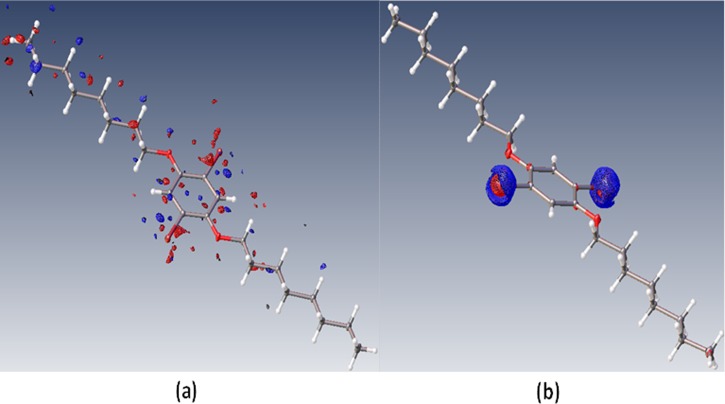
(a) Photoresidual map (F_semi-obs_^ON^- F_calc_^ON^) (isovalues = ±0.30 eÅ^−3^; Blue = positive, Red = negative) and (b) Photodeformation map (F_c_^ON^ − F_c_^OFF^) after refinement of merged data on atomic positions and data-set variables (k_B_, P) (isovalue = ±0.48 eÅ^−3^). A Photoresidual map with isovalues of ±0.36 eÅ^−3^ is shown in Figure S1(c) of the supplementary material.

*Photoresidual maps:* Photoresidual maps are an important tool to judge the reliability and correctness of the refined ES model. They are calculated with the difference between ES structure factors derived from the experimental ratios and the calculated laser-ON models (F_semi-obs_^ON^ − F_calc_^ON^). An example is given below in Fig. 5(a). An additional Photoresidual map is shown in Fig. S1(c) of the supplementary material

*Photodeformation maps*: Photodeformation maps (Fournier and Coppens, 2014 and Jarzembska *et al*., 2014) are calculated using the LASER refined model parameters. They represent the difference between the densities calculated with the excited state parameters and those of the GS structure.

Examples of a Photoresidual map and the Photodeformation map based on the refinement of the merged data are given in Figure 5 below.

Relevant expressions are listed in Section SI-4 of the supplementary material.

#### 2. Least squares refinements

An intensity-ratio (I_ON_/I_OFF_)-based least-squares refinement of atomic positions, excited state population (*P*), and temperature scale factor (*k*_B_) was performed using the program LASER (Vorontsov and Coppens, 2005 and Vorontsov *et al.*, 2010) (see Section SI-3 of the supplementary material for details).

The random distribution (RD) model was employed to describe the excited state structure. It is based on a random distribution of the molecules in the crystal and is generally applicable when conversion percentages are low (Vorontsov and Coppens, 2005). As described above, the shorter data sets 1 and 2 were used to check the quality of the data by plotting the correlation between equivalent reflections in the two sets. Details of all data sets are listed in Table I above.

The refined isotropic temperature scale factors, *k*_B_ = *B*_light on_/*B*_light off_ (Ozawa *et al*., 1998 and Kim *et al*., 2002), are used to estimate the temperature change upon irradiation. They multiply the anisotropic temperature factors obtained before excitation. The initial guesses for the *k*_B_ values at the start of the LASER refinement were obtained from photo-Wilson plots (Schmøkel *et al.*, 2010). Values ranging from 1.13 to 1.20 were obtained for the four data sets and are listed in Table II.

**TABLE II.** Temperature scale factors and excited state populations from LASER refinements. Cut off: ratios with σ(η)/|η|/>0.5 eliminated.

Data set	No. of reflections	Overall completeness (%)	(sin θ/λ)_max_/Å^−1^	*k*_B_ from photo-Wilson plot	*k_B_* from LASER refinement	Excited state Population (*P*) (%)
Laser03	1168	50.3	0.625	1.11	1.13 ± 0.01	2.80 ± 0.90
Laser04	1074	46.3	0.625	1.16	1.17 ± 0.01	4.52 ± 0.73
Laser05	964	41.6	0.625	1.14	1.20 ± 0.01	4.46 ± 0.70
Laser06	941	40.6	0.625	1.13	1.17 ± 0.01	4.99 ± 0.79
Scaled/merged data set	1951	84.0	0.625	1.14	1.18 ± 0.01	5.55 ± 1.09

Two comprehensive refinements were performed. In the first, the four data sets were refined separately in the joint refinement, which resulted in excited state population ranging from 2.80% to 4.99% (Table III, Details are listed in Section SI-3 of the supplementary material, which in Section SI-4 also shows the photo-Wilson plot of the combined refinement, Fig. S1(b)). For the second refinement, individual datasets were scaled and combined as described earlier (Makal *et al.*, 2011) to obtain a single combined scaled/merged data set. It resulted in a 5.55% excited state population (Table II, last row). The relatively low population can be attributed to two facts: first, the laser was operated at a power at which a sufficient amount of data could be collected without damaging the crystal, and second, the quantum yield of luminescence as reported by Shi *et al*. was low (8.9%). Because of the relatively low excited state population and the small increment of the atomic displacement parameters, *U*_ij_, (ranging from 0.0019 to 0.0029Å^2^, upon excitation), the same unit cell dimensions for both laser-OFF and laser-ON structure refinement were used.

**TABLE III.** Bond length changes and atomic shifts upon excitation of independently refined atoms (combined set). See Figs. S1(a) for the direction of the shifts and S1(b) for the Photo-Wilson plot of the merged data, respectively (supplementary material).

Bonds	Bond lengths in GS (Å)	Bond lengths in ES (Å)	Bond length changes (Δd, Å)
Br…Br	3.29000(1)	3.3800(3)	0.0900(3)
C7-Br1	1.886(1)	1.904(1)	0.018(2)
C7-C5	1.395(1)	1.242(3)	−0.153(4)
C7-C1	1.398(1)	1.388(2)	−0.010(3)
C1-O3	1.360(1)	1.427(7)	0.068(8)

The refined excited state geometry shows an expansion of the bond length for C7-Br1 and C1-O3 and contraction for bonds C7-C5 and C7-C1 (Table III). The shift in atomic position is largest for C7 at 0.102(1)Å, followed by C5 (0.069(1)Å), C1 (0.064(2)Å), Br1 (0.0530(1)Å), and O3 (0.029(1)Å), respectively. We note that atoms C1 and C5 are not related by symmetry, C1 being chain-substituted, while C5 is linked to a hydrogen atom, as shown in Fig. 1. In addition, the molecular environment is quite different for the two atoms. The intermolecular Br···Br distance increased from 3.290 Å in GS to 3.380 Å in ES. The position and orientation of the ES species were also refined as a rigid body in the second refinement, leading to small reorientations (<4°, listed in Table SIII of the supplementary material.)

The planarity of the BrC_7_C_5_C_1_O group changes by 0.37 upon excitation. The torsion angle between C_5_C_7_Br and BrC_7_C_1_ changes from 1.30° to 2.72° upon excitation in the crystal.

## IV. QUANTUM CHEMICAL CALCULATIONS

**FIG. 6.**
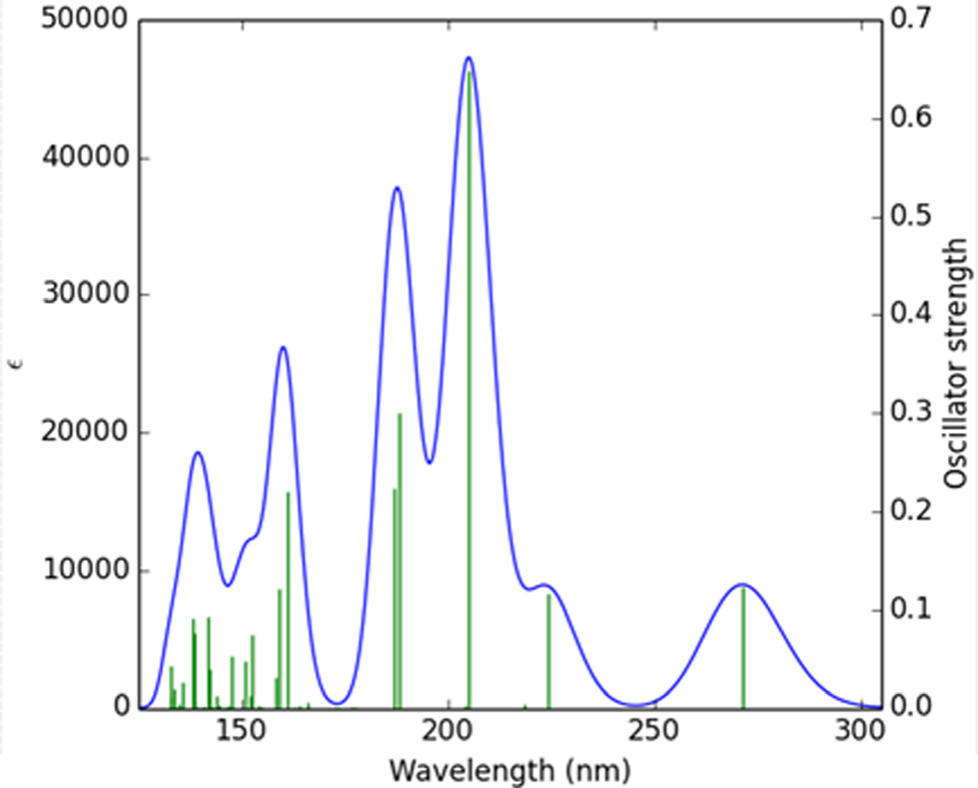
Theoretical HSE06 UV-Vis spectrum.

DFT calculations were performed with the program Gaussian 09 (Frisch *et al.*, 2009) using both the HSE06 (Heyd and Scuseria, 2004a; 2004b; Heyd *et al*., 2005; Krukau *et al*., 2006; Izmayov *et al*., 2006; and Henderson *et al*., 2009) and the PBE0 functionals (Adamo and Barone, 1999 and Perdew *et al.*, 1996). Calculated spectra of the isolated molecule are very similar to the two techniques, with neither extending beyond 300 nm. Both agree on the assignment of the peak at ∼275 nm to a HOMO-to-LUMO transition. The HSE06 spectrum is shown in Fig. 6. The spectroscopic experiment shows that the longest wavelength peak is significantly red-shifted in the crystal, thus explaining the 355 nm excitation. Energies and oscillator strengths of the calculated transition are listed in Table SIV of the supplementary material, while the atomic contributions to the frontier orbitals are given in Table SV.

To obtain information on the effect of the energetic significance of the increase in the Br…Br contact distance, the single point energy for the C-Br…Br-C fragment was calculated at the experimental Br…Br distance, keeping the C-Br distance constant to eliminate effects in other molecular changes. The counterpoise correction (Boys and Bernardi, 1970) was used to evaluate the difference between the energy of the four atom fragment and the sum of the isolated two atom species, thus eliminating the Basis-Set-Superposition-Error (BSSE). The calculations show a decrease in the Br…Br repulsion energy from 202 kJ/mole to 108.8 kJ/mole corresponding to the increase of the Br…Br distance from 3.290 to 3.380 on excitation.

## V. DISCUSSION

Fast non-radiative quenching and high sensitivity towards the surrounding environment have so far restricted the usefulness of organic phosphorescent materials. As described in the introduction to our paper, it has now been shown that organic solids with unusually short Br···Br interactions with phosphorescence lifetimes of milliseconds can be synthesized (Mukherjee and Thilagar, 2015; An *et al.*, 2015; Gong *et al.*, 2015; and Shi *et al.*, 2016). Many of such compounds are described in the literature but have not been examined spectroscopically. Nor have any been subjected to time-resolved (TR) studies which can shed light on the nature of the interaction. We conclude that a relaxation of the Br…Br distance is involved, but the exact nature of the excitation is to be explored in more detail. The crystals contain “infinite” links of Br-Br connected molecules, which raises the question to what extent the excitation is localized.

Our study is to be followed up by synthesis of other compounds in this class and additional theoretical calculations. The crucial questions to be addressed are whether strong luminescence is a general feature of this class of compounds, what restrictions apply, and if the molecules in luminescent crystal can be modified by chemical substitution to manipulate their properties.

## VI. SUMMARY

In the present work, the excitation of the luminescent dibromobenzene derivative, 1,4-dibromo-2,5-bis(octyloxy)benzene, was studied by in-house monochromatic time-resolved diffraction. The results show an increment of the intermolecular 90 K Br···Br contact distance from 3.290 Å to 3.380 Å. They also show an elongation of the C7-Br1 bond length, which increases from 1.886(1)Å to 1.904(1)Å, on laser induced excitation. Calculations show the Br…Br interaction to be strongly repulsive in both the GS and ES states but significantly relaxed by the lengthening of the contact distance on excitation The stability of the crystals is attributed to the many weak C-H…Br and C-H…π intermolecular interactions.

The study described is the first comprehensive application of In-House Time-Resolved diffraction, made possible by the dramatic increase in the brightness of X-ray sources and highly sensitive detectors. As this increase is continuing, many more applications may be expected. Purely organic luminescent materials are particularly suitable for this purpose as they tend to have long phosphorescence lifetimes and are relatively cost effective in industrial applications (Mukherjee and Thilagar, 2015).

## VII. SUPPLEMENTARY MATERIAL

See supplementary material for information on the Spectroscopic Measurements, the Ground State crystal structure, details on the Response Refinement, general expressions for Photoresidual and Photodeformation Map, and details on the Quantum Chemical calculations.
